# Lessons Learned from Battling COVID-19: The Korean Experience

**DOI:** 10.3390/ijerph17207548

**Published:** 2020-10-16

**Authors:** Sang M. Lee, DonHee Lee

**Affiliations:** 1 College of Business Administration, University of Nebraska-Lincoln, Lincoln, NE 68588, USA; slee1@unl.edu; 2 College of Business Administration, Inha University, Incheon 22212, Korea

**Keywords:** COVID-19 pandemic, Korean response strategies, lessons learned, public health systems, equity

## Abstract

Background: The COVID-19 pandemic has swept the world like a gigantic tsunami, turning social and economic activities upside down. Methods: This paper presents some of the innovative response strategies implemented by the public health system, healthcare facilities, and government in South Korea, which has been hailed as the model country for its success in containing COVID-19. Korea reinvented its public health infrastructure with a sense of urgency. Results: Korea’s success rests on its readiness, with the capacity for massive testing and obtaining prompt test results, effective contact tracing based on its world-leading mobile technologies, timely provision of personal protective equipment (PPE) to first responders, effective treatment of infected patients, and invoking citizens’ community and civic conscience for the shared goal of defeating the pandemic. The lessons learned from Korea’s response in countering the onslaught of COVID-19 provide unique implications for public healthcare administrators and operations management practitioners. Conclusion: Since many epidemic experts warn of a second wave of COVID-19, the lessons learned from the first wave will be a valuable resource for responding to the resurgence of the virus.

## 1. Introduction

The recent spread of the coronavirus (COVID-19) has thrown the world into total chaos. COVID-19 has caused not only a health and social crisis of immense proportions forcing people to deal with the fear of infection and the physical, emotional, and financial damage from government recommended physical distancing, but it has also caused a global economic turmoil [1,2]. The virus started spreading in Wuhan, China in late December 2019, and became a shocking pandemic by mid-March 2020; by this time, it had already caused several hundred deaths, disrupted the global economy, and forced countries to close their doors to visitors [3,4,5]. The World Health Organization (WHO) defined COVID-19 as “an infectious disease caused by a newly discovered coronavirus” [6]. WHO declared the novel coronavirus outbreak a global pandemic, the highest category for infectious diseases, on 11 March 2020, officially notifying the global community that a health crisis is upon us [3,7].

Major infectious diseases that have occurred during the past 20 years include Severe Acute Respiratory Syndrome (SARS: 2003), novel influenza virus (H1N1: 2009), Middle Eastern Respiratory Syndrome (MERS: 2012), avian influenza (2014 and 2017), and COVID-19 (2019), most of which were originally reported in China. Particularly, COVID-19 has potent infectivity, implying that it spreads quicker and its mutations are more complex than other infectious diseases. As the numbers of confirmed cases and deaths began to rise exponentially, halting the coronavirus became a global issue. In addition to the immediate health concerns, the COVID-19 pandemic disrupts and destroys global supply chains along the associated systems of purchasing, manufacturing, logistics, and sales [8].

The Global Risks Report 2020, released recently at the World Economic Forum (WEF) in Davos, Switzerland, emphasizes that an infectious pandemic is one of the top 10 risks that could cause a global economic crisis (WEF, 2020a). The report described infectious diseases as “low likelihood, high impact” events, so that when an outbreak occurs it will have a major detrimental impact on the global economy and society. The Global Risks Report further stated that, although many countries have established certain epidemic prevention systems since the Ebola epidemic (which occurred in West Africa in 2014), healthcare systems that need to respond to novel infectious diseases (e.g., SARS, Zika virus, and MERS) are still outdated [9]. No country is perfectly prepared to control an infectious disease, thus collective vulnerability in dealing with the social and economic effects of a pandemic is a global issue. Especially, governments must establish appropriate infrastructures for epidemic emergency management, transparent media for information disclosure regarding the emergency, and call on the collective wisdom of citizens to fight the disease [10,11,12].

In Korea, the information pertinent to COVID-19 has been promptly and transparently disclosed through the Korea Center for Disease Control and Prevention (KCDC), local government websites, and automated text message systems, which communicate in real-time the recent locations where newly diagnosed patients have traversed, thereby encouraging the voluntary civic engagement of citizens (e.g., handwashing, wearing masks, and social distancing). One of the biggest differences between the current pandemic and past similar infectious disease cases is that COVID-19 erupted when digital transformation was in full operation, especially in Korea [13]. Owing to the best information and communications technology (ICT) infrastructure and cloud-based mobile environment in the world, Korea took full advantage of its technological capabilities for managing the spread of COVID-19. For example, Korea introduced an innovative drive-through testing system for massive daily tests, almost instantaneous analysis of tests (within five minutes for the results), mobile technology based geotagging for contact tracing, and the use of online-based remote work activities (e.g., video conferencing, tele-medicine, working from home, and online classes) [14]. Such an advanced digital environment positively contributes to preventing a wide spread of the pandemic in Korea [1,8,15].

The new normal in the pandemic period is truly painful to all people, especially for those who have been engaged in air travel, tourism and hospitality, sports, entertainment, education, and large operating systems. In this new environment, people are advised to avoid direct human-to-human contacts. Thus, the digital infrastructure and operational innovations play renewed significant roles [8,16]. For example, online-ordered deliveries, curbside pickups, and “untact” (no human contact) services are flourishing [17]. With the increased demand for remote and untact services, many innovative apps are also being developed (e.g., apps to locate the nearest place to purchase masks, to have a rental car delivered to the house, to have desired meals delivered in the shortest time, etc.) Specifically, the need for telemedicine has been highlighted as patients with pre-existing conditions or chronic diseases are advised to refrain from visiting their regular healthcare providers due to hospitals over-crowded with coronavirus patients [1].

Currently, COVID-19 has spread across the globe, without showing any sign of abatement as no effective general cure or vaccine for the pandemic is available. The onslaught of virus-infected patients has pushed the healthcare systems of many countries to the brink of collapse [7,18]. In those nations where health systems are still holding on, the prevailing social anxiety pertaining to the stretched capability of medical staff and facilities is extremely high [7,18]. The collapse of a healthcare system refers to the downfall of the first line of defense (e.g., treating a patient effectively until the next patient requires help) in the face of communicable and infectious diseases. Such a situation can paralyze many people with anxiety and fear that their health support system may collapse and not be able to protect them from the pandemic. The current pandemic clearly indicates the need for the public healthcare system to be agile, flexible, and resilient to encounter this emergency health crisis [5,19]. This is precisely the reason that innovative measures must be developed based on the lessons learned from the current and past pandemic crises.

This study attempts to present directions for potential changes in the crisis response systems of public healthcare worldwide, by analyzing COVID-19 pandemic response cases, both successes and failures, in Korea. More specifically, this study has the following objectives: (1) To analyze Korean experiences with cases where healthcare facilities failed to prevent previous infectious diseases from spreading, and how these failures served the government in devising effective approaches to encounter the COVID-19 pandemic, (2) To dissect cases that showed innovative and successful response measures to deal with the COVID-19 pandemic, and (3) To elaborate on suggestions for crisis management based on the lessons learned from these COVID-19 response cases in Korea.

The rest of this paper is structured as follows. In Section 2, we present a review of relevant literature on global infectious diseases and COVID-19 as well as several real cases of Korean healthcare providers in managing the COVID-19 pandemic. Section 3 presents the innovative response strategies deployed in Korea (K-response model) to fight the pandemic. In Section 4, we summarize the important lessons learned from the Korean experience. We conclude the study in Section 5 by discussing implications of the study results, limitations of the study, and future research needs.

## 2. Relevant Literature

### 2.1. Global Infectious Diseases and COVID-19

Infectious diseases refer to those “caused by pathogenic microorganisms, such as bacteria, viruses, parasites, or fungi; the diseases can spread, directly or indirectly, from one person to another” [20]. These diseases can infect people by contact with other humans, animals, or other reservoirs infected by a pathogen or toxic substance. Additionally, communicable diseases refer to those that directly or indirectly spread between humans or between humans and animals. Thus, an infectious disease pandemic is an epidemic that has the potential to be easily transmitted and to affect the global population due to its highly infectious nature [21].

Since the Second World War, the world has seen many innovative developments in vaccines and antibiotics; such advances have ensured that communicable and infectious diseases are reasonably controlled [11]. An important study by the Institute of Medicine, “Emerging Infections: Microbial Threats to Health in the United States,” shed some light on the issues involved with novel infectious diseases [22]. Soon thereafter, the WHO passed the resolution “Global Health Security: Epidemic Alert and Response” in 2001 [23], which enabled the collection of information and enforcement of actions through cooperative work by inter-governmental agencies, non-governmental institutions, private organizations, and governments around the world.

In 2003, the SARS outbreak in China quickly spread fear of a pandemic that could cross borders and affect countries worldwide. Particularly, China failed to promptly and transparently disclose epidemic information. The Chinese government reported the outbreak to the WHO several months after the first confirmed case of SARS, thus delaying effective response measures by world organizations. Immediately, the WHO raised the alert that the SARS outbreak was of high risk, subsequently issuing a travel advisory notice (e.g., advising a travel ban to places where the epidemic had occurred) aimed at suppressing the spread of the disease. As a result of this experience with SARS, many countries worldwide recognized the importance of a global infectious disease governance system, which should stretch beyond the governance of each country [24]. In 2005, the International Health Regulations were revised and expanded to include not only communicable diseases but also other possible threats (i.e., biological terrorism and events that induce international public health crises). Nevertheless, other highly contagious diseases have continued to emerge throughout the last two decades.

In the presence of healthcare emergencies, such as the infectious and coronavirus outbreaks discussed above, public healthcare should be available throughout the country not only with rapid response but also based on an equitable basis [5,19,25]. The sense of equity involves a person’s perception of the input and output relationship which should not create tension or displeasure as a result of cognitive dissonance [26]. Rousseau [27] defined equity as a function of customer’s perception in a service encounter experience. Equity is realized when a person believes his/her outcome concerning resources invested is in harmony with that of others [28]. Equity theory has become the theoretical foundation for service recovery as it helps create possible recovery approaches for service failures through recognition, procedures, and mutual interaction involving customer complaints [29,30]. It is important that people perceive that a service is being provided equitably. Especially in a crisis such as the COVID-19 pandemic, it is imperative for people to perceive that urgent public healthcare is being provided equitably [5]. Such perceived equity inspires people to be transparent about their activities (e.g., infection status, contacts, self-quarantine, etc.) that are the first line defense against the disease. This study examines the successes and failures of the Korean healthcare organizations in their efforts to contain COVID-19, from an equity perspective relating to healthcare services.

### 2.2. Cases of COVID-19 among Healthcare Facilities in Korea

Korea has a history of responding poorly to infectious diseases (e.g., SARS, H1N1, and MERS). In 2012, Saudi Arabia was the first country to experience the MERS outbreak. Korea was the most detrimentally affected country by the virus as many Korean global firms have operations in Saudi Arabia. In Korea, the first MERS case was confirmed in 2015 and its rapid spread resulted in a significant number of casualties which heightened anxiety that swept throughout the country. Furthermore, the serious blow caused to the national economy clearly revealed the weakness of Korea’s infection crisis management system [10,31]. The Korean government’s limited response capacity regarding MERS and its poor communication to its citizens weakened people’s trust in the government’s infection crisis management policies to the point where many started believing that the national epidemic prevention system could easily collapse [31]. There were 185 confirmed cases of MERS among those who traveled to the Middle East, 38 of whom died in 2015. The causes of the high mortality rate could be attributed to the limited capacity of the healthcare delivery system for handling the new virus, shortage of epidemic prevention equipment for medical first responders, and the moral hazard among patients [10,31].

After painful experiences in dealing with past diseases, the Korean government was determined to establish an effective infrastructure to deal with future epidemic emergencies, with KCDC as the control tower. The new infrastructure includes an increased number of negative-pressure isolation wards, real-time systems for data and transparent information collection and analysis, and modernization of the healthcare system. Since the MERS crisis, the Korean government has reinvented a national healthcare delivery system equipped with advanced digital technologies and expanded the facilities specifically designed to deal with infectious diseases (e.g., the creation of negative pressure wards) [10,31].

Thus, KCDC was well prepared to respond effectively to epidemic emergencies when the COVID-19 crisis occurred. When COVID-19 began to spread, the Korean government raised the response level to serious (the highest) on 23 February 2020 and promptly established the Central Disaster and Safety Countermeasure Headquarters, headed by the Prime Minister to bolster government-wide responses to the virus with KCDC as the command center [32].

According to the Korea Economic Daily [33], the rapid spread of COVID-19 around the world, especially in China, Italy, and in the United States, and the subsequent spike in the number of deaths, has brought global attention to the prevention model and early response operational strategy implemented by Daegu city, the epicenter in Korea. Daegu took aggressive actions with speed to prevent the collapse of its healthcare system without placing the city in a lockdown [34]. The city government performed aggressive screening, testing, and quarantining of patients in the communities that were confirmed to have, or suspected of having, infected citizens.

According to KCDC [32], Daegu city did not implement this approach at the onset of the COVID-19 outbreak in Korea (18 February 2020). Daegu and the North Gyeongsang province (where Daegu is located) were heavily criticized for the exponential growth rate of infected patients as ground zero. This region accounted for 70% of the confirmed cases in Korea, caused primarily by the Shincheonji Church gatherings (worship services where people sat on the floor shoulder-to-shoulder) and the mass infections that occurred among the first medical responders while providing care services. The city government performed screening tests of the entire congregation of the Shincheonji Church, isolated severely ill patients, and secured enough quarantine beds for those in need of treatment and isolation. To achieve this, Daegu operated a public-private partnership (PPP) network (composed of the Emergency Response Advisory Group, the Daegu Medical Association, and three infectious disease management support groups), which served as the control team for the COVID-19 epidemic [35].

The PPP collaboration network deployed several response strategies against COVID-19. First, private hospitals were converted into isolation hospitals. A group chatroom for the control team was created, through which experts held discussions about the situation throughout the night. Through these discussions, the Daegu Dongsan Hospital and the Ministry of Defense were contacted and asked to secure as many beds as possible at the Daegu Armed Forces Hospital and Daejeon Hospital. At that time (18–25 February), there were only about 30 available negative pressure wards in Daegu, and some confirmed patients died while waiting to be admitted into a hospital.

Second, the entire congregation of the Shincheonji Church was tested, and those who had symptoms were identified. On the night of 18 February 2020, 70% of confirmed cases of COVID-19 were members of the Shincheonji Church. The Daegu secured information on the 3000 members of the Church, identified them, and ordered 544 symptomatic patients to remain in self-quarantine for two weeks. A leading physician at the Kyungbook National University stated that “the rate of confirmed cases reached 80% among patients who showed symptoms; so, if we had not prompted early isolation of those Shincheonji Church members who showed symptoms, Daegu might have been in the same situation as Europe or the United States” [33].

Third, members of the Daegu Medical Association provided care to those patients in self-quarantine via video calls using 100 outgoing-call-only smartphones provided by Daegu city, hence eliminating the previously existing void in the response system. These response activities represent innovative strategies implemented in the initial stage of the Covid-19 invasion (e.g., aggressive testing, almost immediate test results, contact tracing of infected persons, and prompt treatment of severely ill patients). The mortality rates in New York, USA (6.44%) and in Madrid, Spain (12.62%) are much higher than that in Daegu, Korea (2.76%) as of 1 June 2020 (see Table 1). These high mortality rates indicate that their patient monitoring and healthcare facilities operations were not systematic. It is evident that “the most potent operational strategy amid the lack of a cure is not search and destroy, but identification and isolation of symptomatic citizens” [33].

Fourth, drive-through screening centers were developed for the first time in the world, supported by a world-leading ICT infrastructure [36]. The Yeungnam University Medical Center, in the vicinity of Daegu, had admitted a COVID-19 patient on 19 February 2020, which resulted in the closing of the emergency room (ER) and prompted self-isolation of its medical staff. Based on this experience, the Medical Center decided to establish a drive-through screening center, which eliminated the risk of shutting down ER and self-quarantining first responder medical staff. Moreover, the existing screening center was inefficient in handling the large crowd of people needing testing in a small space. Thus, this was another innovation in need that led to the strategy of developing drive-through testing centers. Laura Bicker, a BBC correspondent in Seoul, referred to the drive-through testing centers as “such a clever idea and so quickly set up”. Sam Kim, an economics reporter at Bloomberg, mentioned that Korea “once again proved to be among the world’s innovative nations,” and Ian Bremmer, president of the Eurasia Group, a think tank in the US, stated that “innovation drives resilience” [37]. Innovative operation strategies are critical in fighting such a formidable global pandemic as COVID-19.

Finally, creative applications of the national ICT infrastructure and rapid development of mobile apps by young entrepreneurs have helped analyze the details about confirmed patients and their contacts (e.g., locations, people contacted, and travel patterns before their infection confirmation). KCDC [32] collected and released relevant information (e.g., regions, pockets of high infection density, and places visited) on COVID-19 patients in real-time. Such transparency regarding the handling of COVID-19 patients encouraged citizens to voluntarily participate in physical distancing and personal hygiene. This is another strategy that has helped Korea effectively manage the crisis when compared to other nations such as Italy and the US. In Korea, after KCDC disclosed the movements of the first COVID-19 patient in Daegu on 18 February, all the places the patient had visited were immediately shut down and disinfected; moreover, the government analyzed security footage (e.g., CCTV from the entrance of the church) to help identify and isolate anyone who might have come into contact with the patient. Local governments sent out emergency alert text messages to provide real-time updated information so that the population of other provinces and cities could be advised not to visit the infected locations.

Table 1 summarizes the current (as of 1 June 2020) state of the cities with the greatest number of confirmed cases among countries most affected by COVID-19, and the innovative operational strategies implemented by Daegu to respond to the COVID-19 pandemic. Daegu, the epicenter of COVID-19 cases, is currently in the process of being transformed into a smart city. It is noteworthy that the city/area where an explosive outbreak of COVID-19 cases occurred had higher mortality rates than that of the country as a whole.

Table 2 summarizes statistics of the number of confirmed COVID-19 infection cases, deaths, and mortality rates among the top 10% of 169 countries, including Italy, China, and South Korea, as of 7 September 2020. The table also indicates average statistics for the entire 169 countries.

**Figure 1:**
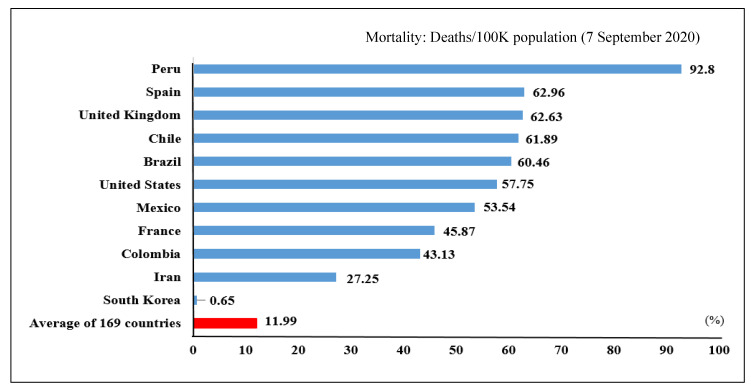
Mortality rates in the most affected countries. Source [45].

Figure 1 shows the mortality rates of the top 10 countries for COVID-19 infection, including South Korea, as of 7 September 2020 (including the average for the entire 169 countries as a group). The bars in the figure show the number of deaths per 100,000 population. In the early phase of the coronavirus spread, South Korea recorded the second highest number of infected persons after China. However, as shown in Table 1 and Table 2 and Figure 1, Korea initiated aggressive strategies for testing and contact tracing based on its well-established public health infrastructure. Thus, the country has been able to flatten the curve of infected cases which resulted in relatively low rates of deaths/infected cases and mortality (deaths/100,000 population) [44].

The crisis caused by a pandemic can lead to issues of equity in the public healthcare service [46]. Particularly, failures in providing equitable public healthcare and in community participation in the decision making process should not be repeated. Thus, it is necessary to analyze the successes and failures experienced in the current situation (i.e., the first wave) as a preparation for the possible onslaught of the second wave of the pandemic. To conduct an in-depth analysis of the causes and consequences of the COVID-19 virus spread in Korea, we examine the operational procedures and strategies implemented by several healthcare facilities. Many seriously ill patients with the virus were diagnosed or infected in ER. Even in a normal day, ER operates at the disaster level [47]. The COVID-19 pandemic severely tested the agility, flexibility, and resilience of ER operations at every hospital. The following five cases are investigated based on the information released by KCDC [32].

#### 2.2.1. Hospital A

Hospital A is an 808-bed tertiary general hospital located in Seoul with 2000 employees. The hospital provides care to an average of 600 inpatients and 2000 outpatients daily. The first COVID-19 case in this hospital was confirmed on 21 February 2020, and was consequently quarantined for two weeks until 5 March. A careful contact tracing of the patient resulted in 14 additional confirmed cases within the hospital. The first confirmed case in the hospital was a patient aide who helped patients move from the ward to the lab. Prior to the diagnosis, it was found that this aide had helped 207 patients. After the diagnosis of the first confirmed case, the hospital’s ER and outpatient clinics were closed and quarantined.

#### 2.2.2. Hospital B

Hospital B is a 251-bed general hospital with 644 employees located in Seoul. It was closed for two weeks, from 8 to 22 March, due to a patient’s dishonesty. The patient was a resident of Daegu City but falsified his home address when hospitalized. After being diagnosed with COVID-19, the patient was isolated. The hospital shut down its outpatient center, some wards, and ER. The particular concern which this patient caused was the fact that the person had visited the artificial kidney unit, contacting many high-risk patients. Although there was no additional positive case found, the hospital was quarantined for two weeks.

#### 2.2.3. Hospital C

Hospital C is a psychiatric hospital (housed on floors 8–11 in a high-rise building) in Daegu with 286 inpatients and 72 employees. The first positive diagnosis of COVID-19 was made on 26 March, after which the number of confirmed cases increased at an alarming rate. This case happened because the first patient was originally at a convalescent hospital (located on floors 3–7 in the same building) where the first positive case was tested on 20 March and spread quickly to133 patients. When the first case was confirmed at the convalescent hospital, the building management firm failed to disinfect the entire building and consequently the virus spread to Hospital C. Hospital C conducted virus tests on its own employees but did not screen all patients because all the employees were tested negative. There were 196 infected patients as of 20 April and the hospital was closed. KCDC conducted a thorough investigation of Hospital C and found that the mass infection occurred because of the failure to screen all patients [32]. Further, due to the nature of the psychiatric hospital (i.e., closed wards in confined spaces), the infection spread rapidly, and subsequently the number of infected cases increased at an accelerated rate.

#### 2.2.4. Hospital D

Hospital D is a 700-bed general hospital located in Seongnam City, in the vicinity of Seoul, with 1400 employees, including 140 specialists in 26 specialty areas. The hospital serves an average of 5000 patients daily. One patient was discharged after treatment in the hospital and returned to receive outpatient care. The same patient became very ill and was brought to ER and diagnosed with COVID-19 infection on 5 March. Consequently, a mass infection of the virus occurred among healthcare providers within the hospital (43 confirmed cases). Hence, the hospital was closed from 6 March to 12 April (38 days). Hospital D provided incomplete information to KCDC, as a person in need of isolation was omitted from the list [32].

#### 2.2.5. Hospital E

Hospital E implemented proactive measures to suppress the spread of COVID-19. All patients and their respective caregivers were required to fill out a paper-based health questionnaire upon admission. However, there were concerns about having the patients complete the questionnaire, which might take too much time while they were crowded in a limited space. In order to reduce crowding and minimize hospital-acquired virus infections, a mobile health questionnaire was delivered by the hospital. The questionnaire asked patients to precisely list all travel to foreign countries, visits to regions or facilities with confirmed cases, and any symptoms of fever or respiratory difficulties.

Hospital E reported that (https://www.yuhs.or.kr/en/), between 12 and 19 March, an average of 6136 people submitted the mobile health questionnaire each day. On average, the questionnaire took 1 min 29 s to complete (8.9 s for each of 10 items). Further, Hospital E sent the mobile health questionnaire to visitors scheduled for outpatient services or testing at 6 AM on the day of the appointment via KakaoTalk (Korean SNS) or text message. Once the patient had completed the questionnaire, a quick response (QR) code was generated. If the patient had self-reported COVID-19-related flagging, a red QR code was assigned, and no flagging was noted with a black QR code. Only visitors who had the red QR code were issued with proper stickers and allowed to enter the hospital.

The patients with the red QR code were required to undergo an additional evaluation at the hospital entrance. Based on this evaluation, they were either directed to a designated safe care facility or were not allowed to enter the hospital for treatment. Moreover, visitors who were not able to use a mobile questionnaire or were not aware of this requirement were provided with the paper-based questionnaire at the entrance. The implementation of this mobile questionnaire was an effective measure to reduce hospital-acquired infections, among the patients and between employees and patients (hospital press release; https://www.yuhs.or.kr/en/).

Table 3 presents a summary of problem causes and response strategies employed by the sample hospitals.

## 3. Korean’s Innovative Response Strategies to the COVID-19 Pandemic

During a pandemic crisis, the government should encourage people to take preventive measures for their own safety, and share information transparently, including that on the risk involved. After failing to effectively manage the MERS outbreak in the past, Korea improved its public health infrastructure and expanded its relevant medical facilities. This experience enabled the Korean government and healthcare providers to develop innovative response strategies to COVID-19.

### 3.1. Innovative Testing System

Drive-through and walk-through testing centers were implemented for the first time in the world. These systems have been heralded as a creative response model to the pandemic, and they have been benchmarked and copied by countries worldwide [14,36,48]. The compelling motivation for these systems was rooted in the challenging problems experienced by some screening centers. These centers were overwhelmed by an onslaught of patients with suspected COVID-19 symptoms. The average waiting time for a screening was as high as six hours or even longer in some cases, consequently raising serious cross-infection concerns [32].

A drive-through screening center can process suspected patients through several steps (e.g., completion of the patient questionnaire, physician’s examination, sample collection, and education) without them getting out of their automobiles, hence shortening the testing duration to less than 10 min per patient [32]. These testing systems lower the risk of infection, minimize contact between test recipients and healthcare providers, and save time.

In a regular screening center, the room must be disinfected and ventilated after treating each patient. Drive-through centers, on the other hand, do not require such procedures, thus not only diminishing the risk of contamination but also greatly increasing the number of daily cases handled. For example, a regular screening center could process two cases per hour (20 daily cases), whereas a drive-through center can process up to six cases per hour (60 daily screenings) [32]. Furthermore, the people being tested preferred the drive-through system because the waiting time for the test is far less and the queue time is spent in the comfort and safety of their own cars [48].

The most challenging task in controlling the spread of COVID-19 is making sure incoming international travelers are not carriers of the virus. Thus, KCDC set up 16 walk-through outdoor screening booths (eight for each of the two international terminals). All incoming passengers are tested as they walk through the station at the rate of 5 min per person, screening more than 2000 passengers per day.

One of the most important aspects of suppressing the spread of the pandemic is the battle against time. Performing aggressive testing in a short period of time to identify and isolate confirmed patients is the most critical operational strategy for flattening the curve of infection cases [49]. The screening systems discussed here represent innovations developed due to the desperate need to contain COVID-19 at the most critical point of time, the beginning of the rapid spread stage [32].

### 3.2. Innovative Approaches to Addressing the Shortage of Healthcare Facilities

On 10 March 2020, the Korean government established residential treatment centers to treat confirmed COVID-19 patients while maintaining their quarantine. In this residential system, each room is occupied by one person as a rule, and all residents must conduct self-monitoring twice daily and are on constant monitoring and treatment by health professionals residing in these centers. Such centers’ primary aim is to triage and categorize patients into four levels (mild, moderate, severe, critical), and the secondary aim is to manage patients with mild conditions. This system helps ensure that hospital beds are provided for COVID-19 patients needing critical inpatient care.

The resident treatment centers are managed independently by each local government. The primary goal of these centers is to prevent the spread of the virus by treating, isolating, and medically monitoring infected patients. While it is not necessary to send patients with mild symptoms to hospitals, it is still necessary to isolate them as they have the potential to widen the spread of the disease. Thus, these patients are the major targets of residential centers. To achieve their strategic objectives, these residential centers need to make their operations flexible and dichotomous, encompassing both hospitals and centers. Hence, their provision requires accurate triage of confirmed patients, active participation by residents, and strict compliance with the care policies and regulations within the center. These residential treatment centers represent an innovative model of care that has helped prevent the spread of the infection to the community while addressing the shortage of hospital beds.

### 3.3. Expansion of “Untact” (Non-Face-to-Face) Services

“Untact” means no human-to-human contact in service encounters [17]. During the COVID-19 pandemic, Korea has been able to provide untact services to its population based on innovative applications of digital technologies. This operational strategy has contributed a great deal to suppressing the spread of COVID-19 through an advanced healthcare system (e.g., drive-through screening centers). One of the best examples of such untact services is the self-diagnosis app and the self-quarantine protection app [32].

On 10 February 2020, the Central Disaster Management Headquarters of Korea announced that it was going to use a self-diagnosis app to monitor the virus infection status of all incoming travelers through a special entry procedure in order to strengthen infection management. This app monitors the symptoms once a day and provides a quick consultation by medical staff for everyone who enters the country. After this app became mandatory, permission to enter Korea has been granted to only those (citizens and foreigners alike) who provided their personal information (e.g., name, passport information, nationality, and actual address) in the app. Anyone entering Korea must consent to use the app and download it themselves as a special entry procedure. The results of the self-diagnosis feature (i.e., fever, cough, and/or sore throat) are submitted to the corresponding public health center and KCDC. Moreover, this app provides important information to the user about screening centers (i.e., the closest available area/location for inspection and contact information), the SNS channel of KCDC, and 1339 consultation call centers.

Untact technology-based approaches are currently making a substantial contribution to address the challenges regarding patient management, shortage of healthcare staff, and lack of resources needed to provide a diagnosis. Additionally, aimed at healthcare providers, a dashboard was developed by the Korea University Medical Center and Softnet to automatically send information about patients’ (self-assessed) body temperature, symptoms recorded on the app, pulse and blood pressure (taken with a Bluetooth blood pressure cuff) to healthcare providers [50]. The application of such innovative apps involves a trade-off between the concern of staying safe from the virus and personal privacy [50]. Nonetheless, these measures have proven to be essential for preventing the spread of the pandemic through prompt and convenient reporting of symptoms among all travelers to Korea.

### 3.4. Strategies for Pandemic Management

The crisis caused by a pandemic requires a robust response system to prevent global health, economic, and social disasters. The key lesson learned from the Korean experience in managing the COVID-19 crisis is the importance of innovative response strategies at the onset of the pandemic. Based on the review of some of the experiences of sample hospitals and the practices that have been proven effective, an innovative pandemic management system should include the following strategies.

#### 3.4.1. Plans, Procedures, and Control

The pandemic response cannot be made by one independent organization. There should be a collaborative public-private partnership that can utilize the synergistic capabilities of governments, private enterprises, healthcare institutions, university research centers, and volunteer organizations (e.g., Daegu’s Collaborative Network: Emergency Response Advisory Group + Daegu Medical Association + Daegu Center for Infectious Disease Control and Prevention). Such a collaborative innovation partnership is imperative to generate creative strategies, operational plans, and detailed work procedures.

#### 3.4.2. Governance

When a collaborative system is established, especially with many volunteer entities, it is difficult to seamlessly execute strategies and actions in a timely fashion in the face of the rapidly spreading pandemic. Thus, an explicitly designed governance system with clearly defined roles, responsibilities, and authorities is as important as the strategic plans.

#### 3.4.3. Integration of Healthcare Delivery Systems

In the exponential increase stage of the pandemic infection, healthcare facilities in the ground zero area could be overwhelmed by severely ill patients, causing the collapse of the system. For effective response management, healthcare facilities in the affected area (cities and surrounding counties) should be integrated as an emergency response system. Then, the various healthcare facilities can be dichotomized based on their scale and core competencies so as to designate some as intensive care facilities where severely ill patients are quickly assigned for treatment and others as safe facilities where patients with non-virus related illnesses are treated [4].

#### 3.4.4. An Effective Logistics System

Fighting a pandemic requires much more than simply testing and treating infected patients. It is essential to secure a sufficient quantity and quality of medical supplies, protection of frontline health professionals, and secure supply chains. It is imperative to develop an effective logistics system for timely delivery of medical supplies (e.g., testing kits, medications, ventilators, additional ER facilities, ambulances, helicopters, etc.). The healthcare delivery system is bound to collapse if frontline healthcare providers are infected because of insufficient or ineffective personal protective equipment (PPE) such as face shields, masks, and body covers (for head, hands, shoes, and body). In addition, stable and emergency supply chains are necessary to ensure timely supply of all medical and other supplies in order to provide urgent care to the patients on an on-demand basis in the face of the pandemic crisis [8].

#### 3.4.5. Devolution of Control and Execution

The spread of a pandemic is not even throughout a country or region. While a national pandemic management center is necessary (e.g., KCDC) to develop nation-wide guidelines and strategies, each region of the country has its own unique patterns of virus infection. Thus, each local area government or virus control center should be allowed to establish its own strategies to control the spread of the pandemic. As the virus spreads throughout the community at a rapid pace, the need for mature civic consciousness usually grows proportionately. The current physical distancing campaign is a good example. Many countries incurred enormous damage due to citizens not abiding to recommended guidelines to prevent the spread of the pandemic (e.g., social distancing, shelter-in-place, personal hygiene, etc.), awakening the need for social co-consciousness and citizen engagement [51]. Therefore, policymakers of the central government should develop definite policies regarding the devolution of control and direction, which are required of all citizens during the critical phase of a pandemic.

## 4. Lessons Learned from COVID-19

The COVID-19 pandemic has brought a total upheaval to the way people live, businesses operate, and governments function. The pandemic has infected more than 27 million people, brought profound sadness to people who lost their loved ones (881,464 deaths as of 7 September 2020), and hundreds of millions lost their jobs around the world in a matter of several months. We must learn from our experience of failure, and some successes, so that we can be better prepared to prevent and manage future pandemics. There are several lessons that have now been learned and identified as important elements for managing a pandemic from the COVID-19 experience in Korea.

### 4.1. Government’s Response Capacity

At the time of the MERS outbreak, the Ministry of Public Safety and Security of Korea sent out tips to the entire population on how to prevent the virus via an emergency text message. However, this only occurred during the early days of the outbreak, and the government’s crisis management system—which should have directed efforts to manage the epidemic—failed to function properly [10]. Consequently, despite having a response system for infectious diseases, the government failed to effectively execute early responses and was criticized for aggravating the damage caused by MERS. This inefficacy of early responses resulted in human casualties, public anxiety, and substantial economic damage, all of which revealed the weakness of the government’s infection crisis management system [52]. This failure not only made the Korean government recognize the vulnerability of the national epidemic prevention system but also provided an opportunity to learn from the failure [31].

Leadership was recognized as a key contributor to the successful containment of the SARS outbreak in Korea in 2003. Previous studies show that leadership has played a significant role in the effective implementation of proactive measures and facilitation of inter-agency communication in the face of an epidemic [53]. More specifically, during the current pandemic outbreak, the national crisis management capacity which was strengthened due to the lessons learned from MERS helped execute necessary response steps at the critical early stage of the pandemic spread. An emergency leadership team should be established with experts in the relevant fields to develop specific strategies for prevention, response measures, and medical treatment procedures to contain the pandemic.

The international media have been reporting Korean strategies and practices for controlling COVID-19 as a model [44,54]. Most Korean provincial governments have been effectively containing the spread of the pandemic without shutting down business operations such as restaurants, coffee shops, retail stores, and even golf courses [48,54]. Particularly, the international media praised Korea’s testing capacity with its innovative drive-through and walk-through stations, rapid processing for results (less than 4 min), and the Korean government’s information management that did not require a lockdown enforcement like Wuhan, China. The New York Times [55] reported that, if South Korea succeeds in containing the infection as it has, it will set an example for the world to follow. Johnson et al. [56] also noted that Korea’s advanced diagnostic capability was proven to be the enabler of its massive testing of more than 35,000 a day, while the U.S. was testing a mere 426 persons (reported on 25 February 2020). Moreover, Health Korea News [57] reported that the mortality rate of COVID-19 patients was only 0.7% among 10,000 infected patients during the early stage of COVID-19 spread. It is evident that the Korean government’s quick response capacity, information disclosure transparency, and implementation of innovative models to protect patients and healthcare providers were the key factors for early success in containing the pandemic.

Korea had a bitter experience with MERS which helped the government to learn from failures and then establish a proven infrastructure to handle the spread of pandemics. A set of best practices, what is now known as “the K-response model,” is based on standardized proven systems that can be applied by all healthcare providers, including testing procedures, contact tracing, isolation by self-quarantine, hospitalization, treatment procedures of severely ill patients, and release after being cured. The control tower in central government and in each local government should proclaim a standardized preventive system (e.g., closing all large audience events such as sports, theaters, shopping centers or educational institutions; limiting the number of people in any group gathering to 10 or less, with social distancing of 6 feet or more; the stay-at-home policy; closing all business operations for 2–4 weeks, including all personal services such as hair salons, massage parlors, physical therapy centers, dental offices, etc.) [32]. The operating systems and strategies that the Korean government implemented to contain COVID-19 have proven to be effective.

### 4.2. Information Sharing and Utilization of Digital Devices

The Ebola outbreak in West Africa in 2014 caused more than 10,000 deaths with a 40% mortality rate. The affected governments’ approaches to combatting the virus failed to foster trust among the citizens, causing fear, and inaccurate information led to a considerable amount of time spent on tracing the actual movements of those infected [58]. A previous study analyzed the outbreak of novel infectious diseases since 1990, focusing on global pandemic management systems [11]. This study stressed the importance of transnational monitoring and information sharing about the spread of diseases (e.g., disease symptoms and infected areas). Another study investigated the outbreak of SARS and pinpointed the Chinese government’s failure to provide effective early response to the pandemic, either concealing or underreporting, as the reason for the global spread of the disease [59].

The Korean government, in response to the current COVID-19 pandemic, has been reporting the number of confirmed cases, the number of deaths, and the actual movements of confirmed patients in real-time. Such government efforts for transparency and urgency regarding the pandemic have gained the public’s attention regarding the risk involved. Such government efforts also gained the trust of citizens and helped people comply with issued guidelines. The government also announced disinfection measures and schedules for locations where confirmed patients made contact with others on a daily basis. This information indirectly advised the general public not to visit those hot spots. In addition, local governments sent out text messages to all citizens upon confirmation of a new infection case in their region and other helpful safety tips about the virus.

Such real-time information sharing about the pandemic has been possible because of the advanced mobile technology infrastructure and the public’s high mobile device usage in Korea. As COVID-19 began to spread throughout the country in February 2020, various apps were developed rapidly by young entrepreneurs (e.g., apps showing confirmed patients’ locations, the closest place where masks and gloves can be purchased, assistance tips for self-quarantine, etc.). Currently, several European countries (e.g., UK and Italy) are also attempting to utilize a GPS tracking system to locate confirmed patients and to inform disinfection and prevention activity areas using smartphone apps. A team of medical researchers at Oxford University in England published a report suggesting that communicable diseases can be controlled effectively if many utilize digital contact tracing [60].

### 4.3. Community and Civic Consciousness

Based on the current COVID-19 situation in Korea, while the government’s response capacity is crucial to prevent the disease from spreading, mature civic consciousness is essential to ensure social compliance with the imposed measures. In Korea, people did not engage in panic buying during the early days of the pandemic outbreak. For example, when there was a shortage of masks, instead of the profiteering behavior of some hoarders, many people began to donate some of their daily allotted masks to the community for those in need. Mass media also encouraged beneficence through public emotions showing a strong spirit of community and unity. People recorded their daily lives in SNS, including health status and places visited, to help prevent the infection from spreading to others around them. Some people even traveled only on foot to prevent spreading the disease in the community. Furthermore, people complied with the five-day rotating purchase system for masks, which was instituted by the government to ensure a fair distribution to everyone in the face of mask shortages.

The global media has praised Koreans for their voluntary civic engagement and compliance with government guidelines to contain COVID-19. The Washington Post [61] reported that Korean citizens canceled major events and most religious services were held online at the outset of the pandemic breakout. Daegu, the epicenter of a massive spread of the virus, was able to manage the situation without a lockdown, as people in other parts of the country voluntarily refrained from visiting the city. Moreover, the BBC News [62] stated that South Korea was able to manage the spread of COVID-19 without implementing a complete lockdown or strict measures against people’s movement. Koreans voluntarily wore masks everywhere outside of their homes and were tested for COVID-19, demonstrating mature community and civic consciousness. Responding together and responsibly to the threat of COVID-19 have now become a battle cry for Koreans [63].

Contrastingly, there were cases where civic duty was not practiced. There were incidents where people lied about their addresses, pretending to be from an area of mass infection, to receive priority care. There were also cases where people under the required self-quarantine violated the isolation guidelines and roamed around the community restaurants and coffee shops, thus spreading the infection [32]. There was a major relapse of the virus infection after two weeks of almost no daily infection had been reported in Korea. During 24 April–6 May 2020, over 5500 young party goes visited several night clubs in Seoul during the social distancing enforcement period. These clubs are known for their loud music, dancing, and drinking in a rather confined space. These clubs often restrict entry only to young people, enforced by a reverse carding system (usually only under 40 years of age). Among those who visited the clubs, more than 270 infected people were identified by June 1. However, there was a social stigma issue (regarding the sexual orientation of many regular customers of the clubs) involved which made contact tracing difficult for those who visited the clubs. It was reported that many club goers falsified their names or addresses (e.g., cell phone numbers).

To suppress the spread of a dangerous pandemic, a spirit of unity and shared purpose is required. Korean people realized the potentially devastating chain of infection that could sweep through their communities and the country as a result of the misguided actions of a single person. In addition to the government’s control measures, the public’s strict adherence to government guidelines and voluntary participation in implementing certain rules (e.g., mask rationing) based on a sense of community have played a major role in suppressing the spread of COVID-19 [32,62,63,64].

### 4.4. Innovative Application of Technologies

In response to the COVID-19 pandemic, physical distancing has been encouraged based on the recommendation of the WHO [2,32,64]. As people refrain from engaging in outside activities, many businesses (e.g., restaurants) start to experience financial difficulties. The drive-through COVID-19 screening model has been applied to other businesses, and a new drive-through shopping model emerged. For example, South Korea’s large seafood markets are utilizing their parking lots to provide drive-through services, where customers can order sushi meals from their cars as they approach the market and vendors fulfill the order immediately. Department stores are delivering pre-ordered products to customers at the valet parking service lot. In addition, services such as drive-through book-lending and agricultural product sales have flourished [65].

Most of package and food delivery services have transitioned from personal service to the “untact” method that minimizes direct human-to-human contacts. Classes and lectures in elementary, middle, and high schools and colleges have transitioned to online platforms. Video conferences and home-offices have also become the common method of running operations. Automobile repair services now provide a “special pick-up and delivery” option to ensure that their services are untact, helping those customers who have hesitated to visit a service center due to COVID-19.

Diebner et al. [66] pp. 3–4 stated that digital delivery has become a necessity for “most customers who are confined at home” and “that app downloads and new sign-ups have grown between 80–250%” during the COVID-19 pandemic. As COVID-19 has been reported to infect people via contact with infected people’s droplets [32,60], untact services utilizing innovative technology applications have flourished and are expected to expand continuously.

## 5. Conclusions

When a pandemic outbreak occurs, identifying the source of infection and suppressing its spread are the most important steps. Amidst the COVID-19 crisis, healthcare institutions are like battlefield military units that are fighting an enemy with necessary weapons, albeit in the form of much-needed medical supplies. In response to the pandemic emergency, many organizations have shifted to remote-working to ensure operational continuity and employee safety. However, many business firms that cannot operate remotely (e.g., manufacturing plants, construction sites, sports events, etc.) had to completely shut down business. Enterprises are scrambling to make fast adjustments to their disrupted supply chains [8]. Educational institutions were ordered to shift the teaching mode from the classroom to the online educational environment. These are “new normal” in the COVID-19 crisis [2].

This study reviewed the cases of innovative responses, as well as failures, to the explosive spread of COVID-19 in Korea since the first confirmed case on 18 February 2020. Based on the review of these cases, we summarized the lessons learned from Korea’s COVID-19 experiences. The knowledge gained from the struggle against the virus provides new insights about required strategies for managing the pandemic. Our study suggests that the healthcare policy makers and related organizations must be transparent in demonstrating to the citizens that emergency healthcare services are being provided on an equitable basis throughout the country. Based on these experiences, policy makers should develop strategies that include the government’s response capacity, information sharing, mature sense of unity and community, and application of advanced technologies in the time of urgency.

### 5.1. Implications of the Study Results

The results of this study provide several theoretical and practical implications. First, the COVID-19 outbreak taught the world that massive and rapid testing is essential to identify infected patients and infection clusters to prevent the pandemic from spreading. The identified patients can be either treated promptly (severely ill cases) or quarantined. Second, we showed that the spread of an epidemic can be effectively suppressed only through well prepared public health infrastructure; coordinated and exhaustive efforts of the central/local governments, disinfection and prevention authorities and healthcare providers; and the spirit of unity and community of citizens (e.g., adhering to the government guidelines regarding social distancing, stay-at-home, avoiding large gatherings of people, etc.). Third, innovative operational strategies should be established based on past experiences (e.g., the MERS failures) in order to ensure success in managing the pandemic. In Korea, the primary cause of the early spread of COVID-19 was related to a mass gathering within a confined indoor space (e.g., worship services of religious organizations). The Koreans learned quickly about the perils of such undisciplined activities and their consequences in terms of the uncontrollable spread of the pandemic [32].

Moreover, the Korean government enforced an aggressive COVID-19 screening program to promptly identify and trace contacts made by infected people and treat seriously ill patients while strictly isolating them from the general population. Furthermore, these measures cannot be implemented successfully without active cooperation of the citizens. From the outset, the government asked all citizens to refrain from participating in group gatherings or events, both indoors and outdoors, that could pose a threat to others, and strongly encouraged the practice of physical distancing. Moreover, the government also placed a legal liability on agents who proceeded with non-recommended events. A mature civic consciousness is needed to voluntarily comply with government guidelines.

A crisis is said to be a combination of danger and opportunity. President John F. Kennedy analyzed the word “crisis” in Chinese and pointed out that the word consists of two characters, one representing danger and the other representing opportunity [67]. Winston Churchill’s famous quote was also in the same vein, “A pessimist sees the difficulty in every opportunity; an optimist sees the opportunity in every difficulty” [68]. These quotes serve as reminders that every crisis encompasses opportunities for creating a better future. The Koreans have learned this lesson from their experience with the COVID-19 pandemic. Therefore, we might view the crisis from the perspective of “crisis = danger + opportunity” based on response efforts.

The COVID-19 crisis shows how each country organizes the delicate balance between achieving efficient results (avoiding high rate of mortality) and intrusion on personal privacy and economic security. This means that there is a trade-off relationship between two important factors in life: health and economy. For example, much of the offline education system will most likely transition to the online environment, causing a trade-off relationship between students’ face-to-face education needs and a safer/cheaper mode of delivery.

The measures undertaken by the Korean government to avoid repeating the same mistakes incurred during the MERS outbreak (i.e., re-organization of the KCDC, the healthcare delivery system, and disinfection and prevention systems, as well as the expansion of healthcare facilities) were shown to have a significant impact on the effectiveness of the implemented response strategies in the face of the COVID-19 pandemic. Therefore, developing an effective public healthcare infrastructure and new operational strategies based on past experiences could turn a crisis into an opportunity for preventing such virus infections [5]. We are confident that the fear of COVID-19 that is currently sweeping the globe will soon be overcome and hope that this costly experience will serve the world well in preparing for the next pandemic.

### 5.2. Limitations and Future Research Needs

This study has reviewed the response strategies of Korea in dealing with the COVID-19 pandemic outbreak. Korea’s pandemic management approach, known as the K-response strategy, has been effective in containing COVID-19 as the country learned a bitter lesson from the pains of MERS and reinvented its public health infrastructure as a preparation for the next pandemic. We do hope that the operational strategies of Korea discussed in this study would help prepare effective crisis management systems in other nations. This study, however, has some limitations. First, the scope and experience of the COVID-19 cases discussed in this study are specific to Korea. Thus, the results of the study have limited generalizability to other situations or countries with different cultures and social systems. Future research that includes various cases from around the world would further reinforce the findings of our study. Second, the COVID-19 pandemic is still causing havoc around the world and its end is difficult to predict. Furthermore, the world will surely encounter new coronavirus pandemics in the future. The results of our study are limited to the discussion of COVID-19 that we are battling today. Thus, new pandemics will require different research approaches, although the lessons learned from the current pandemic will certainly be of much value.

## Figures and Tables

**Table 1:** Cities most affected by COVID-19 and Daegu’s strategies to combat the pandemic.

Major City (Country)	Number of Confirmed Cases	Number of Deaths	Mortality Rate	Response Strategies Implemented by Daegu to Deal with the Pandemic
Worldwide	6,263,632	375,542	6.00%	Converted private hospitals into isolation hospitals Identified members of Shincheonji church who had symptoms via phone surveys and screening tests Provided 100 smartphones to civilian physicians to assist them in diagnosing, via video calls, patients that needed to be self-quarantined The first city to implement drive-through screening centers (Chilgok Kyungbook National University Hospital, Yeungnam University Medical Center) The first city to implement mobile screening centers (management of high-risk groups such as Shincheonji cluster/nursing home residents) Ran 15 residential treatment centers in collaboration with the government
New York (US): From 20 Jan. 2020	373,040 (1,811,360)	24,023 (105,165)	6.44% (5.81%)	
Madrid (Spain): From 31 Jan. 2020	68,852 (239,638)	8691 (27,127)	12.62% (11.32%)	
Lombardy (Italy): From 31 Jan. 2020	89,205 (233,197)	16,143 (33,475)	18.10% (14.35%)	
Hubei (China): From 31 Dec. 2019	68,135 (84,154)	4512 (4638)	6.62% (5.51%)	
Daegu (Korea): From 20 Jan. 2020	6885 (11,541)	188 (272)	2.76% (2.36%)	

As of 1 June 2020 (unit: person), Sources [38,39,40,41,42,43].

**Table 2:** A summary of COVID-19 statistics for selected countries (7 September 2020).

Ranking Based on Confirmed Cases	Country	Confirmed Case	Deaths	Death/Case	Mortality: Deaths/100k Population
1	United States	6,276,365	188,941	3.00%	57.75
2	India	4,204,613	71,642	1.70%	5.30
3	Brazil	4,137,521	126,650	3.10%	60.46
4	Russia	1,022,228	17,768	1.70%	12.30
5	Peru	683,702	29,687	4.30%	92.80
6	Colombia	666,521	21,412	3.20%	43.13
7	South Africa	638,517	14,889	2.30%	25.77
8	Mexico	634,023	67,558	10.70%	53.54
9	Spain	498,989	29,418	5.90%	62.96
10	Argentina	478,792	9859	2.10%	22.16
11	Chile	422,510	11,592	2.70%	61.89
12	Iran	386,658	22,293	5.80%	27.25
13	United Kingdom	349,500	41,640	11.90%	62.63
14	France	347,268	30,730	8.80%	45.87
15	Bangladesh	325,157	4479	1.40%	2.78
16	Saudi Arabia	320,688	4081	1.30%	12.11
17	Pakistan	298,903	6345	2.10%	2.99
19	Italy	277,634	35,541	12.80%	58.81
35	China	90,058	4730	5.30%	0.34
75	South Korea	21,296	336	1.60%	0.65
Average of 169 countries		160,365	5227	2.91%	11.99

Source [45].

**Table 3:** Example hospitals with confirmed COVID-19 cases.

Case	Cause	Response	Lesson
Hospital A	Noncompliance with regulation by a patient aid	Emergency room and outpatient center closure Quarantined for two weeks Testing of inpatients and employees Employees were allowed to use only personal vehicles to commute	Staff must comply with regulations (mandatory mask use)
Hospital B	False statement by a patient	Hospital closure Testing of all who came into contact with the patient	The need to take responsibility if a public problem occurs (owing to the patient’s false statements) and to have mature civic consciousness
Hospital C	Blithe response	Hospital closure Testing of all patients and employees	The need to test the whole area that was potentially infected (in this case, the entire building)
Hospital D	Secondary mass infection following initial patient infection	Hospital closure Testing of all who came into contact with the patient Staff self-quarantine	The need to ensure data and information transparency The need for full cooperation with the Korea Center for Disease Control and Prevention (KCDC)
Hospital E	Implemented proactive measures	Required a paper-based health questionnaire Sent KakaoTalk or text message with the health questionnaire	To reduce crowding and minimize hospital-acquired virus infections, various measurement methods were used by the hospital
